# Developing a Motor Imagery-Based Real-Time Asynchronous Hybrid BCI Controller for a Lower-Limb Exoskeleton

**DOI:** 10.3390/s20247309

**Published:** 2020-12-19

**Authors:** Junhyuk Choi, Keun Tae Kim, Ji Hyeok Jeong, Laehyun Kim, Song Joo Lee, Hyungmin Kim

**Affiliations:** 1 Division of Bio-Medical Science & Technology, KIST School, Korea University of Science and Technology, Seoul 02792, Korea; h14505@kist.re.kr; 2 Center for Bionics, Biomedical Research Institute, Korea Institute of Science and Technology, Seoul 02792, Korea; ktkim@kist.re.kr (K.T.K.); t20193@kist.re.kr (J.H.J.); laehyunk@kist.re.kr (L.K.); 3 Department of Brain and Cognitive Engineering, Korea University, Seoul 02841, Korea

**Keywords:** hybrid BCI, EEG, motor imagery, FBCSP, lower-limb exoskeleton

## Abstract

This study aimed to develop an intuitive gait-related motor imagery (MI)-based hybrid brain-computer interface (BCI) controller for a lower-limb exoskeleton and investigate the feasibility of the controller under a practical scenario including stand-up, gait-forward, and sit-down. A filter bank common spatial pattern (FBCSP) and mutual information-based best individual feature (MIBIF) selection were used in the study to decode MI electroencephalogram (EEG) signals and extract a feature matrix as an input to the support vector machine (SVM) classifier. A successive eye-blink switch was sequentially combined with the EEG decoder in operating the lower-limb exoskeleton. Ten subjects demonstrated more than 80% accuracy in both offline (training) and online. All subjects successfully completed a gait task by wearing the lower-limb exoskeleton through the developed real-time BCI controller. The BCI controller achieved a time ratio of 1.45 compared with a manual smartwatch controller. The developed system can potentially be benefit people with neurological disorders who may have difficulties operating manual control.

## 1. Introduction

Brain–computer interface (BCI) technology benefits people suffering from neurological disorders on account of its characteristics of various computer-controlled applications using brain signals [1,2]. The recent development of a lower-limb exoskeleton is significant, considering the fact it effectively bridges between brain signals and a motor output of extremities to improve the quality of life of the gait disabilities [3,4,5]. Among the various electroencephalogram (EEG) neural features, three distinguishable ones have been adopted notably for decoding lower-limb movement intentions, namely movement-related cortical potential (MRCP), steady-state visual evoked potential (SSVEP), and event-related desynchronization (ERD). However, utilizing the MRCP for the exoskeleton control requires the BCI system to discern a movement onset time [6]. In the case of the SSVEP [7], subjects have to continuously focus on a flickering light until the evoked potential exceeds a threshold. Thereby, it is difficult for the exoskeleton drivers to deal with an unexpected outer situation. Fundamentally, the ERD is another representative EEG neural feature for the exoskeleton BCI controller, usually induced by motor imagery (MI). An asynchronous MI-based ERD indicates both spectral and spatial features. Hence, the BCI controller can match various commands related to distinctive MI strategies with separable scalp topographic patterns [8].

In the very beginning, project DARPA tried to move prosthetics based on the sensorimotor signals of the cortical activity [9,10]. Additionally, the former EU project named MINDWALKER proceeded lower-limb exoskeleton for clinical use with EEG and various biological and kinematic control signals through advanced algorithms [11,12]. The underlying studies adopted MRCP, SSVEP, and evoked potential (EP) to control robotic devices. Lately, several research groups have reported tenable results in operating an overground lower-limb exoskeleton with the MI-based BCI [13,14,15,16] Gordleeva et al. developed an exoskeleton control system utilizing three MI tasks (left, right hand MI, and rest) and subsequently captured the ERD of sensorimotor rhythms (SMR) for 14 subjects [13]. Lee et al. captured an EEG power spectral density during the hand MI and rest and performed exoskeleton mounted navigation tasks with five subjects [14]. Wang et al. compared an SSVEP and an MI-based BCI controller to move the lower-limb exoskeleton with four subjects and revealed that both controllers achieved about 80% accuracy [15]. Yu et al. developed an MI-based ERD decoder that could control the walking speed of a rehabilitation exoskeleton on the treadmill [16]. However, the aforementioned studies still adopted the left and right (or both) hand MI to generate a corresponding command output for controlling the lower-limb exoskeleton. To our knowledge, there were a few pieces of research inducing a gait-related MI [17,18,19]. Firstly, Do et al. adopted a kinesthetic MI (KMI) to refine motor skills in sports science and cognitive neurophysiology [17]. Lopez et al. considered it as a motor-attempt to move subjects’ right leg as if they have started walking [18]. Finally, Donati et al. trained spinal cord injury (SCI) patients with kick imagery during a rehabilitation program [19]. Notably, it is still considered that previously mentioned MI protocols focused on the fragments of gait motions. Hence, presenting a limited correlation between the imagery and the execution, and only utilized a neural mechanism that is discriminative at a cortical level. Therefore, MIs for operating the overground lower-limb exoskeleton throughout an entire ‘sit-to-sit’ scenario should be more intuitive and associated with stand-up, gait-forward, and sit-down, which may reduce a cognitive load and increase decoding accuracies [20].

A real-life MI-based BCI controller for the lower-limb exoskeleton should maintain a low false activation rate in order to ensure the reliability of a control system. A ‘brain switch’ is a representative concept necessary for the asynchronous BCI to determine whether an ongoing continuous EEG signal implies the user’s intention or not [21,22,23,24,25]. Pfurtscheller et al. demonstrated that the on/off switch utilizing a foot MI-induced beta Event-related Synchronization (ERS) rebound measured from a single vertex channel prevents the false activation of an SSVEP interface [26]. Yu et al. extracted a subject’s voluntary successive eye-blink signal from an ongoing EEG signal from two prefrontal channels to activate/deactivate a P300-based speller [24]. Notably, Ortiz et al. recently introduced an attention level monitor parallel with an MI gamma-band SMR, which detects a subject’s presence or absence of an MI intention [25]. Based on previous researches, this study monitored EEG artifact from an electrooculogram (EOG) signal to extract a user’s intentional triple eye-blink (TEB) signals to turn on and off the MI decoder under a concept of a sequentially processed hybrid BCI for improving reliabilities of the control system [27].

Thus, in this study, we developed an MI-based BCI controller for a lower-limb exoskeleton to perform stand-up, gait, and sit-down, sequentially combined with an eye-blink switch considering a real-life scenario. The feasibility of the developed BCI exoskeleton system was tested with ten healthy subjects to explore the potentiality of its application to people with neurological impairments. This study mainly aimed to reduce a variation between the MI manner and motor output of the mounted exoskeleton. To accomplish this, we designed intuitive MI protocols, which correspond with the lower-limb exoskeleton operation.

## 2. Methods

### 2.1. System Overview

**Figure 1:**
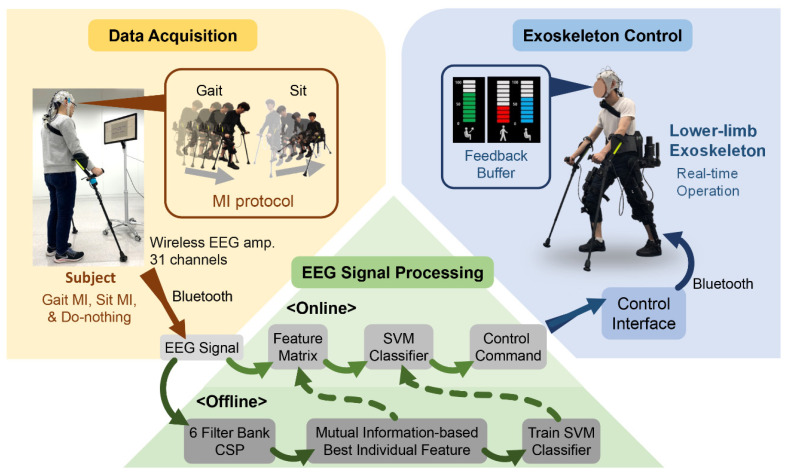
A diagram of the motor imagery (MI)-based brain-computer interface (BCI) exoskeleton control system.

The developed MI-based BCI exoskeleton control system consists of three parts, namely data acquisition, EEG signal processing, and exoskeleton control (Figure 1). While the subject performs MI tasks (i.e., the kinesthetic feeling of gait and sit), a signal processing algorithm extracts features and trains the offline classifier. A decoded control command is sent to the exoskeleton via a real-time online control interface. We employed a lower-limb exoskeleton robot (RoboWear P10, NT Robot, Seoul, Korea) to integrate the developed BCI controller. The exoskeleton robot was primarily designed to assist people with SCI gait impairments (Class III Medical Device Certification, Ministry of Food and Drug Safety of Korea) to stand-up, sit-down, and gait-forward with two crutches on both hands [28].

### 2.2. Data Acquisition

#### 2.2.1. EEG System

Throughout the entire experiment, brain activity was monitored by a wireless wet-type 31 electrodes according to the international 10–20 system (FP1, FP2, F7, F3, F4, F8, FC5, FC3, FC1, FC2, FC4, FC6, C3, C1, Cz, C2, C4, CP5, CP3, CP1, CP2, CP4, CP6, P3, P1, Pz, P2, P4, O1, Oz, and O2. The reference electrode is FCz and the ground is AFz). Each electrode collected brain signal at a 500 Hz sampling rate through an EEG amplifier (actiCHamp and MOVE, BrainProducts GmbH, Gilching, Germany). The impedance level was set below 20 KΩ, and a notch filter cleared 60 Hz line noise.

Ten healthy subjects (age: 26.6 ± 3.06 years.) with no history of neurological disorders participated in this study. The subjects were all male and right-handed. All subjects gave written informed consent, which was approved by the Institutional Review Board of Korea Institute of Science and Technology (KIST IRB number 2019-032). Eight out of 10 subjects had no prior experience in BCI or wearing a powered gait assistive device. We allowed the subjects a one-hour adaptation period to familiarize themselves with operating the wearable exoskeleton.

#### 2.2.2. MI Protocol

**Figure 2:**
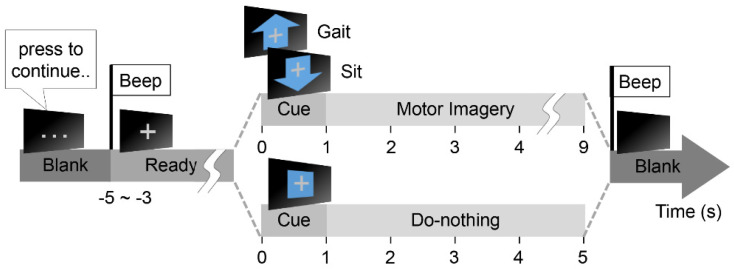
The offline MI procedure.

To minimize external interference, the MIs were performed in an isolated room. The subjects are standing with their hands-on crutches without wearing the lower-limb exoskeleton and facing a monitor, which displayed MI procedures (Figure 1). The subjects were to press a hand-held button attached to the crutch when they were ready to begin each trial. Following the notification of a beep sound, the monitor displayed a gray fixation cross and randomly presented a symbol (‘upward arrow,’ ‘downward arrow,’ or ‘box’) after 3–5 s, which denotes ‘Gait MI,’ ‘Sit MI,’ or ‘Do-nothing,’ respectively. Once the subjects identified the cue, they started the corresponding MI (‘Gait’ or ‘Sit’) for 8 s or ‘Do-nothing’ for 4 s. When the subjects heard a second beep sound, they stopped the task and prepared for the subsequent trial. Figure 2 shows the MI procedure.

Each subject executed two types of MI tasks (‘Gait’ and ‘Sit’) along with a ‘Do-nothing’ task. In the ‘Do-nothing’ task, we let subjects rest with their eyes open without performing MI or other mental tasks. The subjects were instructed during the MI tasks to perform a mental rehearsal of gait or sit. The limbs were to remain still and they were to focus on the kinesthetic feelings, including a somatosensory sensation and experience of motor execution with the exoskeleton. Furthermore, we forbade subjects from visualizing themselves from the viewpoint of an external observer to limit stimulating their visual cortex. The details of the comments were listed in Table 1.

The offline MI procedure consisted of randomly mixed 90 trials, which constituted 30 repetitions for three tasks; Gait MI, Sit MI, and Do-nothing. The whole process was organized and presented on the monitor by a managing software (E-prime3, Psychology Software Tools, Sharpsburg, PA, USA) with an event marking module (BBTK USB TTL, The Black Box ToolKit Ltd., Sheffield, UK).

### 2.3. EEG Signal Processing

EEG signal processing was conducted using MATLAB software (2017a, MathWorks, Natick, MA, USA), which received data through a TCP/IP connection from Remote Data Access host (Recorder, BrainProducts, Gilching, Germany). The offline MI data features were extracted through a Filter Bank Common Spatial Pattern (FBCSP) algorithm. Through a mutual information-based best individual feature (MIBIF) selection method, we sort contributing features as training input to a linear support vector machine (SVM) classifier.

#### 2.3.1. Feature Extraction

Since we focused on the gait-related SMR feature, we monitored ERD from low mu to high beta EEG frequency bands. EEG signals were passed through the zero-phase Butterworth infinite impulse response (IIR) bandpass filter between high Theta to low Gamma frequency (7–34 Hz). The signals were divided into 6 ranges (filter bank; 7–9, 10–12, 13–15, 16–20, 21–25, and 26–34 Hz) considering the subject-dependent dominant frequency features. Next, six bandpass-filtered EEG data were prepared to derive six different CSP transformation matrices.

The single-trial EEG input signal matrix E (where *N* × *T*; *N* is the number of channels; *T* the number of samples in time per channel) is linearly transformed by projection matrix *W*. The spatially filtered signal *Z* given as

(1) Z=WE

We have decided to choose the first and last two rows of signal Z, which differentiate the most [29]. Therefore, the modified transformation matrix has four rows of six frequency bands and channel columns (24 × 31). Finally, the variance difference maximized EEG signals were then log-normalized [30].

#### 2.3.2. Feature Selection

The 24 features then sorted in descending order following the MIBIF method [30], which determined the priority of the signal contributions of well differentiating the two classes. The mutual information of two random variables defined as,

(2) $$M\left(X;Y\right)=\underset{y\in Y}{\sum }\underset{x\in X}{\sum }p_{\left(X,Y\right)}\left(x,y\right)\;log\left(\frac{p_{\left(X,Y\right)}\left(x,y\right)}{p_{\left(X\right)}\left(x\right)\;p_{\left(Y\right)}\left(y\right)}\right)$$

where $p_{\left(X,Y\right)}$ is a joint probability mass function of X and Y, and $p_{\left(X\right)}$ and $p_{\left(Y\right)}$ are a marginal probability mass function of X and Y, respectively. Here, X is each of 24 features, and Y is the corresponding classifier label Y∈{Gait MI vs. Do-nothihg} or {Gait MI vs. Sit MI}. The first k features are empirically selected according to each subject (k=4~10). Finally, the resulting feature matrix was adopted for training the linear SVM classifier.

#### 2.3.3. Real-Time Decoder

The online and offline decoders were synced in signal processing steps. The real-time input EEG signals were sent to the online decoder in every single packet of 31 channels by 10 data points (500 Hz sampling rate). The decoding algorithm ran every 250 data points (window shift). The pre-trained linear SVM classifier outputted a single control command every 0.5 s with a signal processing window size of 2 s. Then, the control interface received the commands to control the exoskeleton.

### 2.4. BCI Controller

**Figure 3:**
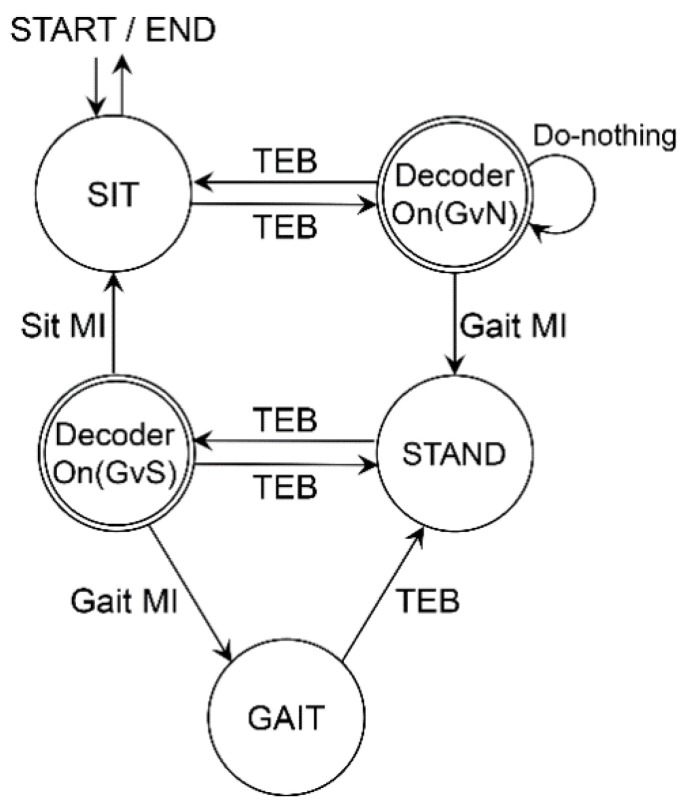
The finite state machine is illustrated in the diagram. The transition between states is indicated in the manner of triple eye-blink (TEB) or MI, respectively. Subjects engage MI in ‘Decoder On’ shown in double circle.

To describe an online system logic flow, we illustrate a finite state machine (FSM) of the control interface (Figure 3). The system should be started and terminated from the sit state for safety purposes. The state transitions were represented by arrows corresponding to methods (MI, Do-nothing, or TEB), denoted beside the arrow. Notably, a recurrent arrow indicated that the system remains in the current state.

We designed two binary classifiers. In the state of ‘Decoder On (GvN)’, the ‘classifier_GvN’ decodes Gait MI vs. Do-nothing EEG signal. In the ‘Decoder On (GvS)’ state, the ‘classifier_GvS’ separates Gait MI vs. Sit MI.

#### 2.4.1. Triple Eye Blink

We utilize TEB (online 97 trials test, a detection rate of 94.7%; online 40.5 min test, FPR of 0.025 times/min; *n* = 1) to activate and terminate the decoder. Notably, a blinking artifact easily influenced two prefrontal channels among the adopted electrode locations in this study. For both FP1 and FP2 electrodes, a 2–15 Hz range of IIR bandpass filter was integrated to clear the signals related to the non-eyelid movement. Subsequently, a biorthogonal wavelet function was adopted to enlarge the eye-blink pulse efficiently. Finally, we could count the wave peak, which exceeded a predefined threshold in separating single or double ordinary occasional eye-blinks. A window size of TEB detection was 1.6 s with a window shift of 0.4 s [31].

#### 2.4.2. MI Buffer and Visual Feedback

**Figure 4:**
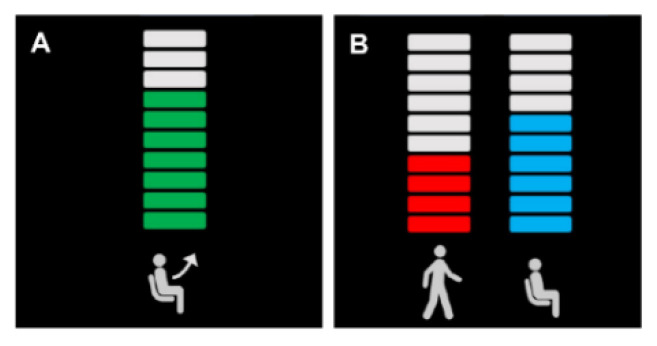
The illustration of the feedback buffer which represented the size of 10 of the exoskeleton BCI controller. (**A**) MI buffer is linked to the classifier_GvN (Gait MI vs. Do-nothing). (**B**) MI buffer is linked to the classifier_GvS (Gait MI vs. Sit MI).

We adopted command stack buffers to minimize potential risks to safety based on a single false detection of the movement intention, as shown in Figure 4. There were three buffers of Sit-to-Stand, Stand-to-Gait and Stand-to-Sit in each size of 10, which is necessary for subjects to engage MI tasks with the exoskeleton movement. First, in the ‘Decoder On (GvN)’ state, the robot stands-up only when the repetitive correct Gait MI command fully filled the Sit-to-Stand buffer, while the Do-nothing command emptied the stacked buffer. Second, in the ‘Decoder On (GvS)’ state, while the Gait MI command filled the Stand-to-Gait buffer, the Stand-to-Sit buffer emptied at the same time, vice versa. The fill/empty ratio of the buffer was set as 1:3 in order to provide reliable state transitions by balancing between the correct and false classification [32].

### 2.5. System Evaluation

#### 2.5.1. Controller Performance

**Figure 5:**
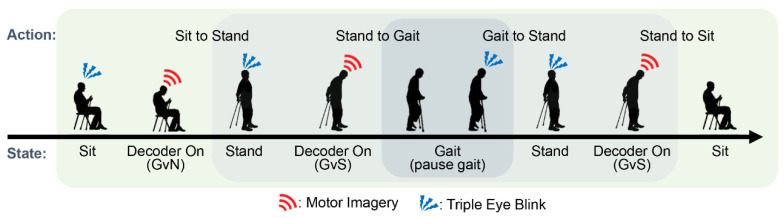
The illustration of the online exoskeleton operation plan. All subjects drove exoskeleton with the scenario of stand-up, gait, pause, resume gait, stop, and sit-down. The subjects completed the procedure two times (one with the BCI controller and the other with the smartwatch controller) to compare the performance of the developed BCI controller and the smartwatch controller.

**Figure 6:**
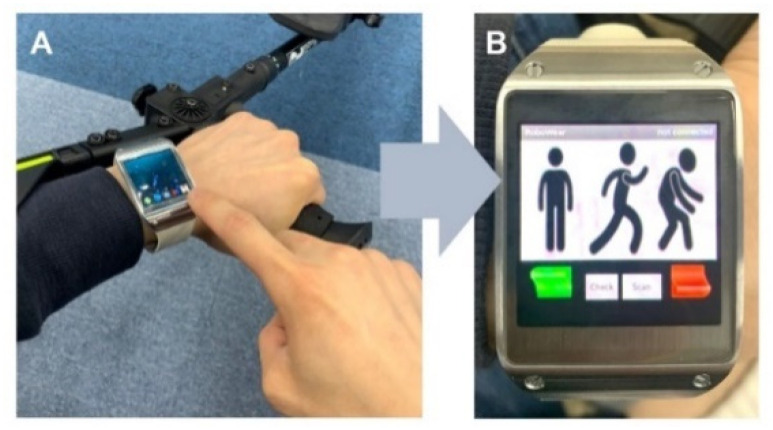
The wearable smartwatch for exoskeleton manual controller (**A**) and the application which was replaced by the BCI controller in this study (**B**).

The online BCI controller was compared with a ready-made smartwatch controller through a predefined 10 m gait scenario to evaluate the developed exoskeleton BCI controller feasibility (Figure 5). All subjects executed stand-up, start 5 m gait and stop, resume 5 m gait and stop again, and finally sit-down. The wearable smartwatch (Galaxy gear series 1, SAMSUNG, Suwon, Korea) and the application were provided to control the exoskeleton (Figure 6). Three control commands (‘stand-up/gait-stop’, ‘gait’, and ‘sit-down’) were transmitted through a Bluetooth wireless communication to the exoskeleton control computer. We compared the required time to complete the gait scenario between the BCI controller and the smartwatch controller.

#### 2.5.2. Classification Accuracy

To evaluate the performance of two binary decoders in offline, we measured classification accuracy of 100 repetitions with the prepared MI data composed with 7:3 of train-test ratio. Initially, randomly chosen trials constituted 10 test questions, and 10 train guesses were sampled by the Bootstrap restoration method except for the test trials. The total result was averaged and reported with a standard deviation.

For the online decoder, we recorded the true positive (TP), true negative (TN), false positive (FP), and false negative (FN) of the two classifiers while subjects were executing the gait scenario. The classifier_GvS showed all four occasions hence the accuracy of the decoder could be calculated. On the other hand, the classifier_GvN operated only a single time during the entire gait scenario. Consequently, we chose to use TPR as an online accuracy measurement of the classifier_GvN.

(3) TPR=nTP/(nTP+nFN)

(4) FPR=nFP/(nTN+nFP)

(5) ACC=(nTP+nTN)/(nTP+nTN+nFP+nFN)

where n stands for the numbers of each of the four parameters: *TP*, *TN*, *FP*, and *FN*. The entire performance of the online decoder was determined as a lower number of the accuracy of two classifiers.

#### 2.5.3. Information Transfer Rate

An information transfer rate (ITR) in communication per unit time was calculated as follows:

(6) $$I_{d}=\log_{2}N+p\log_{2}p+\left(1-p\right)\log_{2}\frac{1-p}{N-1}$$

(7) $$ITR=f_{d}\times I_{d}$$

where $I_{d}$ denotes the bit rate (bit/trial) and N denotes the number of tasks (in this case, N = 3). p denotes decoding accuracy, and $f_{d}$ denotes the decision rate (trial/min) [33]. In the offline session, we assumed the theoretical decision rate as the 90 trial repetitions divided by a total accumulated time of engaging MI for each subject (average of 4.60 trial/min). In the online session, we set the decision rate as an accumulated time of the MI during the entire gait scenario (average of 5.97 trial/min).

## 3. Results

### 3.1. Feature Selection

**Figure 7:**
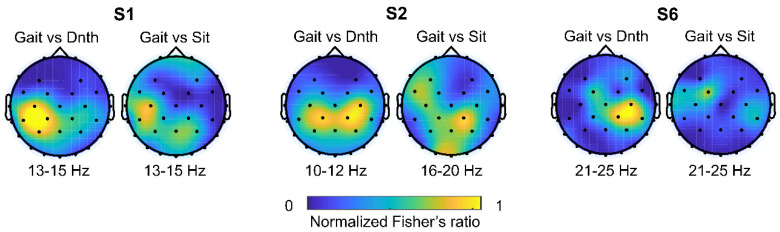
A topography of normalized Fisher ratio between Gait MI(Gait) vs. Do-nothing (Dnth) and Gait MI vs. Sit MI(Sit). Repeated trials of signal power in each frequency band were averaged to calculate the fisher ratio. The most dominant frequency band and electrode channels were visually illustrated and highlighted in yellow color. Three out of ten subjects’ topography were representatively showed to demonstrate a distinct desynchronization area.

**Figure 8:**
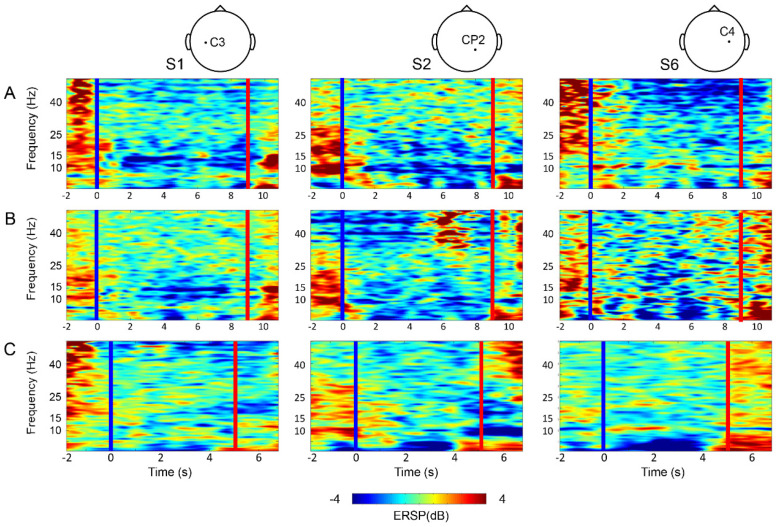
The event-related spectral perturbation (ERSP) of trial averaged power spectrogram plot; (**A**) Gait MI, (**B**) Sit MI, and (**C**) Do-nothing from top to bottom. The blue vertical line (time 0) represents cue onset, and the red line depicts offset. Subjects engage MIs at time 1 to 9 s and Do-nothing at 1 to 5 s.

The MI repetition data was processed to reveal discriminant MI features (Figure 7). Through a Fisher’s ratio topography, we could estimate electrodes with a high signal-to-noise ratio. Based on those representative electrodes, we examined a trial-averaged event-related spectral perturbation (ERSP) spectrogram (Figure 8) [34]. The spectrogram reveals that the ERD appeared while subjects are engaging in both Gait MI and Sit MI, whereas less or no ERD was observed during Do-nothing task.

### 3.2. System Evaluation

#### 3.2.1. Control Performance

Table 2 indicates the time taken to accomplish the 10 m gait scenario of 10 subjects. The hybrid BCI controller showed 144.8 ± 15.12% of average performance in terms of operation time compared to the smartwatch controller. Supplementary Video S1 is provided to compare the consuming time between the smartwatch controller and the hybrid BCI controller.

#### 3.2.2. Classification Accuracy

As mentioned in Section 2.5.2, the accuracy of the 10 decoders for each subject were inspected through 100 train-test repetitions. The classifier_GvN showed 88.4 ± 7.48% accuracy, while the classifier_GvS showed 80.3 ± 6.79% accuracy (Table 3).

**Figure 9:**
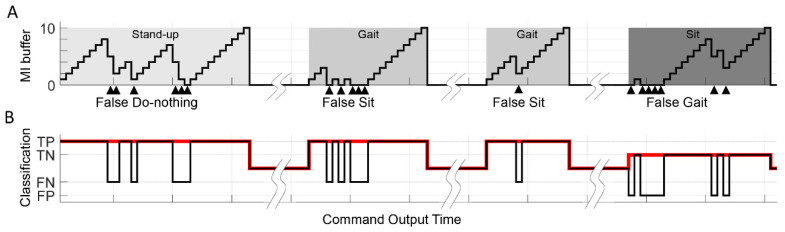
A representative example of the fill/empty log plot of MI buffers (**A**) and MI class discrimination plot (**B**) shares the timeline (subject no. 2). Three kinds of buffers (stand, gait, and sit, size of 10) were illustrated in light-gray, mid-gray, and dark-gray box. Stair shaped line depicts the fill/empty of each corresponding buffer. The false classification was marked as an arrow beneath the timeline. The deviation from true classification (solid red line) was shown in a square plot below.

The online decoder accuracy was estimated by a log record following the execution of the real-time 10 m gait scenario (Figure 9). During the operation, each subject engaged MI for at least four times; (1) to stand-up, do Gait MI for the classifier_GvN, (2) to start gait, do Gait MI for the classifier_GvS, after the TEB (3) to gait again, after gait pause, do same as (2), (4) finally to sit-down, do Sit MI for the classifier_GvS. If the subject failed to fill the corresponding buffer, they made subsequent attempts until they succeeded. The online accuracy was around 85% for both classifiers (Table 3).

#### 3.2.3. Information Transfer Rate

Table 4 shows the ITR for all subjects. By estimating the ITR, we could evaluate the efficiencies of the developed BCI controllers. The offline and online ITR was 3.21 bit/min and 3.13 bit/min on average, respectively.

## 4. Discussion

In this study, we developed an MI-based hybrid BCI controller for the lower-limb exoskeleton operation. The subjects could control the exoskeleton to stand-up, gait start/stop, and sit-down without any steer or button press using the real-time TEB switch and EEG decoder. Ten healthy subjects participated in the offline and online sessions, and the average classification accuracy was more than 80% for both sessions. All subjects completed a 10-m walking scenario with the lower-limb exoskeleton using the MI-based hybrid BCI controller and spent 145% of the control time compared with the conventional smartwatch controller.

### 4.1. Characteristics of the EEG Decoder

As shown in Figure 7, the Gait MI vs. Do-nothing topographic plot appeared relatively consistent through the subjects around a motor and somatosensory area than the Gait MI vs. Sit MI. Following the study of the most prominent electrode channel, we illustrated the MI-related power desynchronization from low Mu (8–12 Hz) to around high Beta (13–30 Hz) frequency band by trial-averaged time-frequency wavelet analysis (Figure 8). The baseline was mean amplitude through the entire epoch time. Within 1 s after the MI cue disappeared, the ERD was revealed in the 10–15 Hz band while few subjects showed EEG signals in the upper bandwidth (21–25 Hz band or higher for S6). According to the research of Cebolla et al., significant ERSP appeared between Mu and low Beta frequency (8 ~ 17 Hz) in FCz channel, induced by the context based MI [35]. Our result also revealed the correlation between MI and spatial-spectral cortical activity on the mu and beta rhythm in the primary motor cortex, consistent with the previous studies [36,37,38]. Additionally, the result demonstrated that the adopted FBCSP algorithm [30] was suitable for incorporating the difference between the Gait MI vs. Do-nothing and the Gait MI vs. Sit MI in terms of both subject-specific spectral and spatial domain.

According to Figure 9A, there were continuous misclassifications. Additionally, subjects experienced a delayed movement of buffer during the MI tasks. The repeated false classification attributed mainly to the EEG processing window set as 2 s length with a 0.5-s window shift. Consequently, if there were a dominant false feature inside the window, it required at least four steps to renew the signal processing window. Moreover, the decoder cannot respond to the subjects’ immediate intention change, consequently allowing a long buffer reaction time. In further research, this problem could be mitigated by shortening the window or reducing the effect of artifacts and noise.

### 4.2. Performance of the BCI Controller

In our study, 10 subjects demonstrated 1.45 of the average time ratio compared with the smartwatch controller. The result suggested that the developed controller could accommodate further improvement. Compared with the existing manual controller, previously developed BCI controllers showed an average time ratio of 2.03 for lower-limb exoskeleton [7], 1.27–1.35 for remote-controlled mobile robots [39,40]. According to the aforementioned studies which presented less performance, considering the subjects were in an ambulatory environment instead of sitting still to control the exoskeleton.

Utilizing the FBCSP algorithm, we could discriminate gait-related SMR with more than 80% accuracy both offline and online. Meanwhile, the classifier_GvN presented an average of 8%-point higher offline accuracy (*t*(18) = 2.6, *p* = 0.018) and 2%-point higher online accuracy (*t*(18) = 0.7, *p* = 0.495) than the classifier_GvS (Table 2). Thus, the EEG feature difference between the Gait MI and the Do-nothing appeared to be more discriminative than the two MIs. Based on interviews of the subjects, we could assess that non-repeating single action imagery such as Sit MI may be less effective in causing the EEG signal variations than the Gait MI, which is relatively familiar and straightforward. This variation might be the reason that the Gait MI vs. Sit MI classification results were not as high as that of Gait MI and Do-nothing despite the instructions and guidelines (Table 1). Further experiments should consider these concerns about the MI protocol.

### 4.3. Limitations and Future Direction

Notably, we acknowledged the existence of numerous alternative novel algorithms for decoding neural features of the EEG signal [41,42,43,44,45]. Among them, deep learning and EEG channel optimization methods are the most relevant methods for this study. Convolutional Neural Network and its applied algorithms are the prominent and spotlighted algorithm for MI signal toward an image domain analysis through the ERSP or short-time Fourier transform (STFT) [43]. Additionally, the EEG MI signals present prevailing spatial feature via a multi-electrodes channel. Consequently, it is recommended to adopt the channel selection method to enhance the performance of the decoder [44]. Further research can proceed from the above-mentioned updating algorithms concerning practical BCI application. While competing with the classification accuracies, in this study, for the first time, we tried to focus on demonstrating the feasibility of the real-time operation of the lower-limb exoskeleton with the gait-related MI accompanied by a conventional yet well-settled FBCSP algorithm. Our approach and findings can form a basis for further developing an online BCI controller for aiding gait disabilities.

Due to the natural and endogenous characteristics of the MI-actuated exoskeleton, it is the most corresponding BCI application to a fundamental property in terms of it’s goal-direct and voluntary nature [3]. Therefore, it is significant that the BCI controlled lower-limb exoskeleton could be advantageous in rehabilitation circumstances [19,46,47,48]. Patients with lower-limb disabilities following a stroke or SCI devote their efforts to regaining the utility of their limbs. The traditional rehabilitation paradigm has been bottom-up, i.e., physical therapists or treadmill move patients’ limb repeatedly to trigger neuroplasticity in the brain. Contrarily, a self-paced assistive exoskeleton controller directly decodes the brain signal and bypasses the path to the damaged limb [49]. Accompanied by this top-down and the classic bottom-up rehabilitation route, a closed-loop feedback interface brings the promising result for the disabilities to regain ambulation ability at will [50,51]. Other researches have also demonstrated the effect of MI-based rehabilitation on balancing or ambulatory skills [19,52]. While this study presents the feasibility of the real-time intuitive MI-based hybrid BCI controller with a wearable exoskeleton on healthy subjects, testing the system with the patients is our intended future study. Further research will recruit more subjects including a SCI gait impairment for practical real-life BCI applications, accompanied by an advanced display device such as portable augmented reality (AR) glasses with an MI assistive environment [53]. We expect that the gait rehabilitation with a BCI-controlled exoskeleton can significantly improve the degree of motor recovery.

## Figures and Tables

**Table 1:** Detail of motor imagery (MI) instructions.

	Operator’s Instructions
Before MI	“Be familiar with consistent locomotion of the robot trajectory with your pair of crutches.” “While practicing ‘sit’, please pay attention to your upper limb movement which plays an important role in lowering the body down to the chair with the exoskeleton.”
During MI	“Pay attention to the kinesthetic sensation that just before your limb about to execute the movement.” “Do mental rehearsal in a slow movement phase, for example, heel strike, weight shift, and toe-off.” “We also recommend you to perceive the input sensation of foot sole and hand grip.” “For ‘Do-nothing’, please ignore the somatosensory or visual input sensation, rather stay unfocused eyes with an absent-minded.”
Prohibited	“Do not picture the scene of observing yourselves or other person’s movement execution.”

**Table 2:** Comparison of operating time between the development hybrid BCI controller and the smartwatch controller.

Subject	BCI Controller (s)	Smartwatch Controller (s)	Time Ratio (%)
S1	170.0	118.6	143.3
S2	125.4	93.7	133.8
S3	145.4	103.2	140.9
S4	159.6	97.1	164.4
S5	144.3	94.2	153.2
S6	157.1	123.7	127.0
S7	153.2	121.9	125.7
S8	138.1	89.5	154.3
S9	180.7	106.6	169.5
S10	158.2	116.6	135.7
mean ± std.	153.2 ± 15.84	106.5 ± 12.84	144.8 ± 15.12

**Table 3:** Offline and online classification accuracy (%).

Subject	Offline		Online	
	GvN	GvS	GvN	GvS
S1	83.3	75.7	94.2	85.9
S2	84.9	77.4	81.3	77.6
S3	80.0	78.4	85.5	85.3
S4	94.0	83.9	81.0	89.2
S5	95.1	74.3	100	86.4
S6	78.0	71.9	88.0	89.5
S7	93.4	79.4	91.7	83.2
S8	98.1	94.4	94.5	88.7
S9	95.1	87.6	78.2	85.9
S10	81.6	77.4	72.2	72.5
Mean ± std.	88.4 ± 7.48	80.3 ± 6.79	86.7 ± 8.61	84.4 ± 5.43

**Table 4:** Offline and online information transfer rate (ITR) (bits/min).

Subject	Offline	Online
S1	1.86	2.99
S2	3.51	2.59
S3	2.71	3.05
S4	3.80	3.31
S5	2.39	3.54
S6	2.24	2.23
S7	2.31	3.64
S8	6.37	3.16
S9	4.80	3.96
S10	2.07	2.80
mean ± std.	3.21 ± 1.442	3.13 ± 0.514

## Supplementary Materials

The following are available online at https://www.mdpi.com/1424-8220/20/24/7309/s1, Video S1.

## References

[1] Pfurtscheller G., Neuper C. (2001). Motor imagery and direct brain-computer communication. Proc. IEEE.

[2] Wolpaw J., Birbaumer N., Heetderks W.J., Mcfarland D., Peckham P., Schalk G., Donchin E., Quatrano L.A., Robinson C., Vaughan T. (2000). Brain-Computer interface technology: A review of the first international meeting. IEEE Trans. Rehabil. Eng..

[3] He Y., Eguren D., Azorín J.M., Grossman R.G., Luu T.P., Contreras-Vidal J.L. (2018). Brain-machine interfaces for controlling lower-limb powered robotic systems. J. Neural Eng..

[4] Tariq M., Trivailo P.M., Simic M. (2018). EEG-Based BCI Control Schemes for Lower-Limb Assistive-Robots. Front. Hum. Neurosci..

[5] Vaughan T.M., McFarland D.J., Schalk G., Sarnacki W.A., Krusienski D.J., Sellers E.W., Wolpaw J.R. (2006). The wadsworth BCI research and development program: At home with BCI. IEEE Trans. Neural Syst. Rehabil. Eng..

[6] Jeong J.-H., Kwak N.-S., Lee M., Lee S. Decoding of walking Intention under Lower limb exoskeleton Environment using MRCP Feature. Proceedings of the GBCIC.

[7] Kwak N.-S., Müller K.-R., Lee S.-W. (2015). A lower limb exoskeleton control system based on steady state visual evoked potentials. J. Neural Eng..

[8] Pfurtscheller G., Lopes da Silva F.H. (1999). Event-related EEG/MEG synchronization and desynchronization: Basic principles. Clin. Neurophysiol. Off. J. Int. Fed. Clin. Neurophysiol..

[9] Miranda R.A., Casebeer W.D., Hein A.M., Judy J.W., Krotkov E.P., Laabs T.L., Manzo J.E., Pankratz K.G., Pratt G.A., Sanchez J.C. (2015). DARPA-funded efforts in the development of novel brain-computer interface technologies. J. Neurosci. Methods.

[10] Vidal J.J. (1973). Toward Direct Brain-Computer Communication. Annu. Rev. Biophys. Bioeng..

[11] Cheron G., Duvinage M., De Saedeleer C., Castermans T., Bengoetxea A., Petieau M., Seetharaman K., Hoellinger T., Dan B., Dutoit T. (2012). From spinal central pattern generators to cortical network: Integrated BCI for walking rehabilitation. Neural Plast..

[12] Wang S., Wang L., Meijneke C., Van Asseldonk E., Hoellinger T., Cheron G., Ivanenko Y., La Scaleia V., Sylos-Labini F., Molinari M. (2015). Design and Control of the MINDWALKER Exoskeleton. IEEE Trans. Neural Syst. Rehabil. Eng..

[13] Gordleeva S., Lukoyanov M.V., Mineev S., Khoruzhko M.A., Mironov V., Kaplan A., Kazantsev V. (2017). Exoskeleton Control System Based on Motor-Imaginary Brain–Computer Interface. Sovrem. Tehnol. Med..

[14] Lee K., Liu D., Perroud L., Chavarriaga R., del Millán J.R., González-Vargas J., Ibáñez J., Contreras-Vidal J.L., van der Kooij H., Pons J.L. (2017). Endogenous Control of Powered Lower-Limb Exoskeleton. Proceedings of the Wearable Robotics: Challenges and Trends.

[15] Wang C., Wu X., Wang Z., Ma Y. (2018). Implementation of a Brain-Computer Interface on a Lower-Limb Exoskeleton. IEEE Access.

[16] Yu G., Wang J., Chen W., Zhang J. EEG-based brain-controlled lower extremity exoskeleton rehabilitation robot. Proceedings of the 2017 IEEE International Conference on Cybernetics and Intelligent Systems (CIS) and IEEE Conference on Robotics, Automation and Mechatronics (RAM).

[17] Do A.H., Wang P.T., King C.E., Chun S.N., Nenadic Z. (2013). Brain-computer interface controlled robotic gait orthosis. J. Neuroeng. Rehabil..

[18] López-Larraz E., Trincado-Alonso F., Rajasekaran V., Pérez-Nombela S., del-Ama A.J., Aranda J., Minguez J., Gil-Agudo A., Montesano L. (2016). Control of an Ambulatory Exoskeleton with a Brain–Machine Interface for Spinal Cord Injury Gait Rehabilitation. Front. Neurosci..

[19] Donati A.R.C., Shokur S., Morya E., Campos D.S.F., Moioli R.C., Gitti C.M., Augusto P.B., Tripodi S., Pires C.G., Pereira G.A. (2016). Long-Term Training with a Brain-Machine Interface-Based Gait Protocol Induces Partial Neurological Recovery in Paraplegic Patients. Sci. Rep..

[20] Talukdar U., Hazarika S.M., Gan J.Q. (2019). Motor imagery and mental fatigue: Inter-relationship and EEG based estimation. J. Comput. Neurosci..

[21] Townsend G., Graimann B., Pfurtscheller G. (2004). Continuous EEG classification during motor imagery-simulation of an asynchronous BCI. IEEE Trans. Neural Syst. Rehabil. Eng..

[22] Han C.-H., Müller K.-R., Hwang H.-J. (2020). Brain-Switches for Asynchronous Brain–Computer Interfaces: A Systematic Review. Electronics.

[23] Han C.-H., Kim E., Im C.-H. (2020). Development of a Brain-Computer Interface Toggle Switch with Low False-Positive Rate Using Respiration-Modulated Photoplethysmography. Sensors.

[24] Yu Y., Liu Y., Yin E., Jiang J., Zhou Z., Hu D. (2019). An Asynchronous Hybrid Spelling Approach Based on EEG–EOG Signals for Chinese Character Input. IEEE Trans. Neural Syst. Rehabil. Eng..

[25] Ortiz M., Ferrero L., Iáñez E., Azorín J.M., Contreras-Vidal J.L. (2020). Sensory Integration in Human Movement: A New Brain-Machine Interface Based on Gamma Band and Attention Level for Controlling a Lower-Limb Exoskeleton. Front. Bioeng. Biotechnol..

[26] Pfurtscheller G., Solis-Escalante T., Ortner R., Linortner P., Müller-Putz G.R. (2010). Self-paced operation of an SSVEP-Based orthosis with and without an imagery-based “brain switch:” A feasibility study towards a hybrid BCI. IEEE Trans. Neural Syst. Rehabil. Eng. Publ. IEEE Eng. Med. Biol. Soc..

[27] Pfurtscheller G., Allison B.Z., Bauernfeind G., Brunner C., Solis Escalante T., Scherer R., Zander T.O., Mueller-Putz G., Neuper C., Birbaumer N. (2010). The hybrid BCI. Front. Neurosci..

[28] Kim Y., Song C., Park J. Development of actuation system for wearable robots using spiral spring. Proceedings of the 2012 12th International Conference on Control, Automation and Systems.

[29] Ramoser H., Muller-Gerking J., Pfurtscheller G. (2000). Optimal spatial filtering of single trial EEG during imagined hand movement. IEEE Trans. Rehabil. Eng..

[30] Ang K.K., Chin Z.Y., Wang C., Guan C., Zhang H. (2012). Filter Bank Common Spatial Pattern Algorithm on BCI Competition IV Datasets 2a and 2b. Front. Neurosci..

[31] Salinas R., Schachter E., Miranda M., Alvarez L., Mejail M., Gomez L., Jacobo J. (2012). Recognition and Real-Time Detection of Blinking Eyes on Electroencephalographic Signals Using Wavelet Transform. Proceedings of the Progress in Pattern Recognition, Image Analysis, Computer Vision, and Applications.

[32] Choi J., Kim K., Lee J., Lee S.J., Kim H. Robust Semi-synchronous BCI Controller for Brain-Actuated Exoskeleton System. Proceedings of the 2020 8th International Winter Conference on Brain-Computer Interface (BCI).

[33] Mcfarland D., Sarnacki W., Wolpaw J. (2003). Brain-computer interface (BCI) operation: Optimizing information transfer rates. Biol. Psychol..

[34] Delorme A., Makeig S. (2004). EEGLAB: An open source toolbox for analysis of single-trial EEG dynamics including independent component analysis. J. Neurosci. Methods.

[35] Cebolla A.M., Petieau M., Cevallos C., Leroy A., Dan B., Cheron G. (2015). Long-lasting cortical reorganization as the result of motor imagery of throwing a ball in a virtual tennis court. Front. Psychol..

[36] Sabate M., Llanos C., Enriquez E., Díaz M. (2011). Mu rhythm, visual processing and motor control. Clin. Neurophysiol..

[37] Stinear C.M., Byblow W.D., Steyvers M., Levin O., Swinnen S.P. (2006). Kinesthetic, but not visual, motor imagery modulates corticomotor excitability. Exp. Brain Res..

[38] Tariq M., Trivailo P.M., Simic M. (2020). Mu-Beta event-related (de)synchronization and EEG classification of left-right foot dorsiflexion kinaesthetic motor imagery for BCI. PLoS ONE.

[39] Millan J.R., Renkens F., Mourino J., Gerstner W. (2004). Noninvasive brain-actuated control of a mobile robot by human EEG. IEEE Trans. Biomed. Eng..

[40] Chae Y., Jeong J., Jo S. (2012). Toward Brain-Actuated Humanoid Robots: Asynchronous Direct Control Using an EEG-Based BCI. IEEE Trans. Robot..

[41] Tang Z., Li C., Sun S. (2017). Single-trial EEG classification of motor imagery using deep convolutional neural networks. Optik.

[42] Roy Y., Banville H., Albuquerque I., Gramfort A., Falk T.H., Faubert J. (2019). Deep learning-based electroencephalography analysis: A systematic review. J. Neural Eng..

[43] Ha K.-W., Jeong J.-W. (2019). Motor Imagery EEG Classification Using Capsule Networks. Sensors.

[44] Jin J., Xiao R., Daly I., Miao Y., Wang X., Cichocki A. (2020). Internal Feature Selection Method of CSP Based on L1-Norm and Dempster-Shafer Theory. IEEE Trans. Neural Networks Learn. Syst..

[45] Jin J., Liu C., Daly I., Miao Y., Li S., Wang X., Cichocki A. (2020). Bispectrum-Based Channel Selection for Motor Imagery Based Brain-Computer Interfacing. IEEE Trans. Neural Syst. Rehabil. Eng..

[46] Lebedev M.A., Nicolelis M.A.L. (2017). Brain-Machine Interfaces: From Basic Science to Neuroprostheses and Neurorehabilitation. Physiol. Rev..

[47] Bockbrader M.A., Francisco G., Lee R., Olson J., Solinsky R., Boninger M.L. (2018). Brain Computer Interfaces in Rehabilitation Medicine. PM R.

[48] Lazarou I., Nikolopoulos S., Petrantonakis P.C., Kompatsiaris I., Tsolaki M. (2018). EEG-Based Brain–Computer Interfaces for Communication and Rehabilitation of People with Motor Impairment: A Novel Approach of the 21st Century. Front. Hum. Neurosci..

[49] Pfurtscheller G., Neuper C., Muller G.R., Obermaier B., Krausz G., Schlogl A., Scherer R., Graimann B., Keinrath C., Skliris D. (2003). Graz-BCI: State of the art and clinical applications. IEEE Trans. Neural Syst. Rehabil. Eng..

[50] Sitaram R., Ros T., Stoeckel L., Haller S., Scharnowski F., Lewis-Peacock J., Weiskopf N., Blefari M.L., Rana M., Oblak E. (2017). Closed-loop brain training: The science of neurofeedback. Nat. Rev. Neurosci..

[51] Morone G., Spitoni G.F., De Bartolo D., Ghanbari Ghooshchy S., Di Iulio F., Paolucci S., Zoccolotti P., Iosa M. (2019). Rehabilitative devices for a top-down approach. Expert Rev. Med. Devices.

[52] Cho H.-Y., Kim J.-S., Lee G.-C. (2012). Effects of motor imagery training on balance and gait abilities in post-stroke patients: A randomized controlled trial. Clin. Rehabil..

[53] Cevallos C., Zarka D., Hoellinger T., Leroy A., Dan B., Cheron G. (2015). Oscillations in the human brain during walking execution, imagination and observation. Neuropsychologia.

